# Genotype-phenotype correlation and description of two novel mutations in Iranian patients with glycogen storage disease 1b (GSD1b)

**DOI:** 10.1186/s13023-019-1266-3

**Published:** 2020-01-31

**Authors:** Maryam Eghbali, Maryam Abiri, Saeed Talebi, Zahra Noroozi, Marjan Shakiba, Parastoo Rostami, Hosein Alimadadi, Mehri Najafi, Fatemeh Yazarlou, Ali Rabbani, Mohammad Hossein Modarressi

**Affiliations:** 1 Department of Medical Genetics, Faculty of Medicine, Tehran University of Medical Sciences, Tehran, Iran; 2 Department of Medical Genetics and Molecular Biology, Faculty of Medicine, Iran University of Medical Sciences, Tehran, Iran; 3 Department of Molecular Medicine, School of Advanced Technologies in Medicine, Tehran University of Medical Sciences, Tehran, Iran; 4 Department of Pediatric Endocrinology and Metabolism, Mofid Children’s Hospital, Shahid Beheshti University of medical sciences, Tehran, Iran; 5 Growth and Development Research Center, Department of Endocrinology, Children’s Medical Center, Tehran University of Medical Sciences, Tehran, Iran; 6 Department of Gastroenterology, Children’s Medical Center, Tehran University of Medical Science, Tehran, Iran

**Keywords:** GSD1b, Autozygosity mapping, Novel variants, Genotype-phenotype correlation

## Abstract

**Background:**

Glycogen storage disease (GSD) is a rare inborn error of the synthesis or degradation of glycogen metabolism. GSD1, the most common type of GSD, is categorized into GSD1a and GSD1b which caused by the deficiency of glucose-6-phosphatase (*G6PC*) and glucose-6-phosphate transporter (*SLC37A4*), respectively. The high rates of consanguineous marriages in Iran provide a desirable context to facilitate finding the homozygous pathogenic mutations. This study designates to evaluate the clinical and genetic characteristics of patients with GSD1b to assess the possible genotype-phenotype correlation.

**Results:**

Autozygosity mapping was performed on nineteen GSD suspected families to suggest the causative loci. The mapping was done using two panels of short tandem repeat (STR) markers linked to the corresponding genes. The patients with autozygous haplotype block for the markers flanking the genes were selected for direct sequencing. Six patients showed autozygosity in the candidate markers for SLC37A4. Three causative variants were detected. The recurrent mutation of c.1042_1043delCT (p.Leu348Valfs*53) and a novel missense mutation of c.365G > A (p.G122E) in the homozygous state were identified in the *SLC37A4*. In silico analysis was performed to predict the pathogenicity of the variants. A novel whole *SLC37A4* gene deletion using long-range PCR and sequencing was confirmed as well. Severe and moderate neutropenia was observed in patients with frameshift and missense variants, respectively. The sibling with the whole gene deletion has shown both severe neutropenia and leukopenia.

**Conclusions:**

The results showed that the hematological findings may have an appropriate correlation with the genotype findings. However, for a definite genotype-phenotype correlation, specifically for the clinical and biochemical phenotype, further studies with larger sample sizes are needed.

## Background

Glycogen storage diseases (GSDs) comprise a heterogeneous group of rare inborn errors of metabolism disorders caused by deficiency of the specific enzymes in glycogen degradation and synthesis. Depending on the impaired enzyme and affected organ, GSDs are classified into over 10 types. GSD1 is the most common liver impairment with an overall incidence of about 1:100,000 live births. It is categorized into 1a (GSD1a) and 1b (GSD1b). GSD1a is the more frequent type responsible for > 80% of GSD 1 patients [1] while GSD1b is estimated to represent ~ 20% of cases [2].

Deficiency in glucose-6-phosphatase-α (G6Pase-α)/glucose-6-phosphate transporter (G6PT) complexes causes GSD1. This complex has a key role in maintaining glucose homeostasis through glycogenolysis and gluconeogenesis in liver, kidney, and intestine. G6Pase-α which is encoded by the *G6PC* gene catalyzes the hydrolysis of glucose-6-phosphate (G6P) to glucose and phosphate. The products will be transported into the lumen of the endoplasmic reticulum from the cytoplasm by G6PT (encoded by the *SLC37A4* gene) [2, 3]. GSD1a disease is caused by the deficiency of the G6Pase-α which is located at the endoplasmic reticulum membrane. GSD1a and GSD1b patients represent similar metabolic phenotypes such as hypoglycemia, hepatomegaly, lactic acidemia, hyperlipidemia, and nephromegaly. Additionally, GSD 1b patients exhibit neutropenia and impaired neutrophil function; resulting in recurrent bacterial infections, inflammatory bowel disease (IBD), and aphthous stomatitis [4]. Nevertheless, not all GSD 1b patients manifest neutropenia, it may cause by either one or more modifiers on G6PT function or *SLC37A4* mutations with residual transport activity [4, 5].

The diagnosis of GSD1 is based on the clinical symptoms, biochemical parameters, and G6Pase activity on liver biopsy tissues which is an invasive procedure. The clinical manifestations are not always useful to differentiate between GSD types 1a and 1b patients [6]. The definitive diagnosis of the disease is established by molecular analysis of *G6PC* and *SLC37A4*.

The estimated rate of consanguineous marriages in Iran is about 38.6% [7] which provides an appropriate context for autozygosity mapping. This powerful tool helps us to quickly identify the possible defective gene flagged by autozygous blocks. Accordingly, in this project we identified candidate short tandem repeat (STR) markers flanking *G6PC* and *SLC37A4* with acceptable heterozygosity in the selected population. Suitable families who showed autozygosity for the markers flanking *SLC37A4* were selected for additional molecular genetics investigations. The aim of this study is to evaluate the clinical and genetic characteristics of patients in order to assess the possible genotype-phenotype correlation.

## Material and methods

### Patients

Patients were recruited from the Children Medical Center Hospital and the Mofid Children’s Hospital in Tehran, Iran during January 2015–April 2019. Twenty Iranian patients from 19 unrelated families were investigated. Inclusion criteria were based upon clinical presentations of hepatomegaly, “doll face” and biochemical laboratory tests such as hypoglycemia, hypertriglyceridemia, hypercholesterolemia, hyperlactatemia, hyperuricemia, and elevated aspartate aminotransferase (AST) or alanine transaminase (ALT) suggestive of GSD.

The other laboratory measurement was cell blood count including neutrophil count and white blood cell count (WBC). In addition, patients were selected after confirmation by histological analysis of liver biopsy. All patients participating in this study had consanguineous parents. Genetic counseling was conducted and all subjects and/or their parents signed the consent form. The Ethical Committee of the Tehran University of Medical Sciences approved the study.

### Molecular genetics studies

**Fig. 1:**
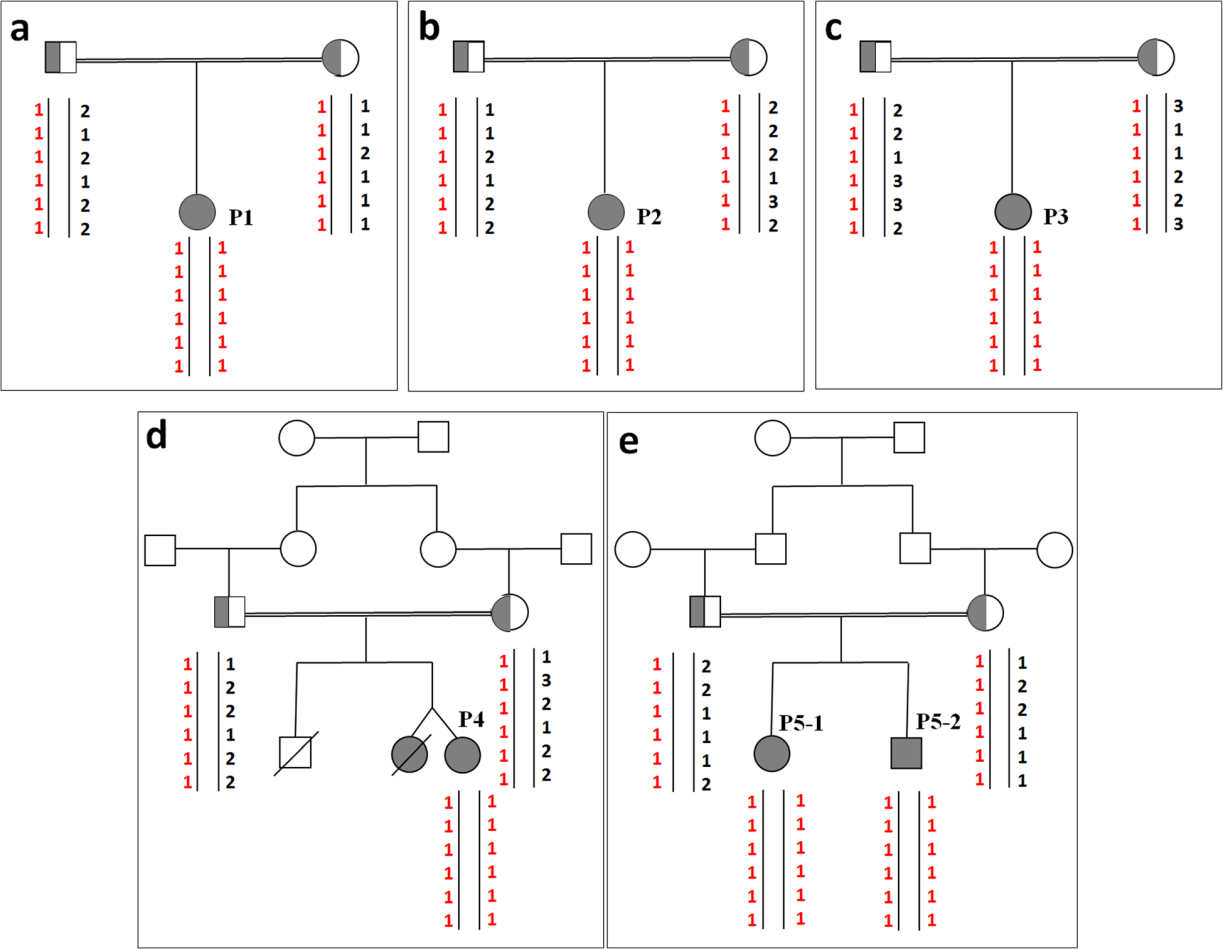
It shows haplotype analysis of the investigated families. **a**, **b** & **c** The affected Childs (P1, P2 & P3) showed autozygosity for STR markers flanking of *SLC37A4* gene which mutation analysis revealed c.1042_1043delCT mutation. **d** The affected child (P4) showed autozygosity for STR markers flanking of *SLC37A4* gene which mutation analysis revealed c.365G > A mutation. **e** The affected siblings (P5–1 & P5–2) showed autozygosity for STR markers flanking of the *SLC37A4* gene which mutation analysis revealed a large deletion

Human genomic DNA was isolated from peripheral leukocytes using Salting out method [8]. To indirectly find the possible mutated gene, autozygosity mapping was performed using the proper STR markers flanking *SLC37A4* and *G6PC* genes. Six polymorphic STR markers linked to these genes were selected using Tandem Repeat Finder (TRF) and Sequence-based Estimation of Repeat Variability (SERV) software [9, 10]. An attempt was made to select markers with the length of 3–5 nucleotide repeats, high allelic heterogeneity and the nearest markers flanking upstream and downstream of the genes responsible for GSD1. The heterozygosity of the selected markers was assessed in 10 random and unrelated individuals. The selected markers were amplified with specific primers (the primer sequences are available upon request). Then the PCR products were analyzed by running on 10.0% polyacrylamide gels and silver nitrate staining. Then a haplotype map was drawn for each family (Fig. 1). Then exons and intron-exon boundaries of the candidate gene were sequenced (the primer sequences are available upon request) and were compared with the cDNA reference (NM_001164277 and NM_000151). PCR reaction for sequencing was performed in a total volume of 25 μl which included 8 μl the Taq 2× Master Mix (Amplicon Company), 1 μl of each 10 pM primer, 13.5 μl DH2O and 1.5 μl of 50 ng/μl DNA. Amplifications of all the exons were carried out under the following program; 95 °C for 5 min, 95 °C for 30s, 60 °C for 30s, and 72 °C for 40s repeated by 35 thermal cycles using a thermal cycler (Applied Biosystems, USA) and final step at 72 °C for 5 min. The novel mutations were named according to the nomenclature recommendations of the Human Genome Variation Society (HGVS) (http://www.hgvs.org).

### Long-range PCR assay

To identify the presence of whole *SLC37A4* gene deletion and to confirm the deletion breakpoint sequences, three primers (F1, R1, and R2) were designed using Primer 3 software for two Long-range PCRs and Sanger sequencing. The sequences and chromosome position of these primers were shown in Table 1. In this Long-range PCR assays, the 25 μl PCR mixture contains 12.5 μl of LongAmp Taq 2X Master Mix (New England Biolabs), 0.5 μl of each 10 pM primer, 10 μl DH2O and 1.5 μl of 50 ng/μl DNA. The first Long-range PCR assay was carried out under the following program; 94 °C for 30s, 94 °C for 30s, 62 °C for 60s, and 65 °C for 8 min repeated by 30 amplification cycles and final step at 65 °C for 10 min. The second Long-range PCR assay was performed with the slightly different program; 94 °C for 30s, 94 °C for 30s, 62 °C for 50s, and 65 °C for 1.5 min repeated by 30 amplification cycles and final step at 65 °C for 10 min. The PCR products were run using 0.8% agarose gel electrophoresis.

**Table 1:** The primers characteristics and the size of PCR products used for Long-range PCR assays

	Name	Sequence	Chromosome Position (GRCh37)	PCR product size
First long-range PCR	F1	AGCATCACTACTGTTACTCCTCAC	Chr11: 118902469–118902492	8276 bp
	R1	GGAGAATGCTGACCCTGATGAC	Chr11: 118894217–118894238	
Second long-range PCR	F1	AGCATCACTACTGTTACTCCTCAC	Chr11: 118902469–118902492	2724 bp
	R2	ATGTCCATGGATCTCAGAGCTTC	Chr11: 118899769–118899791	

### In silico assessment of pathogenicity of the novel variants

Pathogenicity of the variants was investigated using several criteria: (1) population databases such as 1000 genome project (1000 GP) (http://browser.1000genomes.org), dbSNP (http://www.ncbi.nlm.nih.gov/snp), Exome Aggregation Consortium (ExAC) (http://exac.broadinstitute.org), NHLBI GO Exome Sequencing Project (ESP) (http://evs.gs.washington. Edu) were investigated to assess allelic frequency of the variant. (2) in addition, ClinVar (http:// www.ncbi.nlm.nih.gov/clinvar), HGMD (http://www.hgmd.org), Ensemble (https://www.ensembl.org) and recently published articles on PubMed was searched for previously reported variants. (3) A variety of in silico tools consisted of PROVEAN [11], PolyPhen-2 [12], MutationTaster [13], HOPE [14], Combined Annotation Dependent Depletion (CADD) [15], and DANN score [16] were used to evaluate the functional effect of the novel variants on the protein. The multiple tools such as PhyloP and PhastCons through UCSC genome browser and GERP were used to investigate the conservation scores [17]. (4) For further confirmation of the pathogenicity of the variants, segregation analysis of the parents was performed using direct sequencing. (5) The identified variants were classified and interpreted according to the ACMG-AMP 2015 Standards and Guidelines [18] that facilitated by the Varsome tool [19].

## Results

### Patient phenotypes

The clinical, biochemical, and hematological parameters of patients with detected causative variants of the *SLC37A4* gene are presented in Table 2. There were significant differences in the clinical and biochemical parameters indicating heterogeneity between these GSD1b patients. Consanguinity was detected in all cases. Almost all patients presented hepatomegaly and hypoglycemia. Also, all patients had recurrent infections including otitis, respiratory tract infection, gingivitis, oral candidiasis, pharyngitis, periodic aphthous stomatitis. Hematological findings were different in patients; P1, P2, and P3 with severe neutropenia, P4 with moderate neutropenia and a sibling (P5–1 and P5–2) with both severe neutropenia and leukopenia (leukocyte count and percentage of neutrophil cells were shown in Table 2). Anemia was observed in all patients except for P4. The other main biochemical parameters were hyperlipidemia, hyperlactatemia, and elevated AST and ALT levels (four patients (66%)), hypercholesterolemia (two patients (33%)), hyperuricemia (three patients (66%)). The clinical information collected from the mentioned patients (P5–1 and P5–2) belongs to the time of disease diagnosis.

**Table 2:** Table caption

Patient no.	P1	P2	P3	P4	P5-1	P5-2
Mutation						
cDNA	c.1042_1043delCT	c.1042_1043delCT	c.1042_1043delCT	c.365G>A	g.118895235_118901	g.118895235_118901
Protein	p.Leu348Valfs*53	p.Leu348Valfs*53	p.Leu348Valfs*53	p.Gly122Glu	946del	946del
Gender	F	M	F	F	F	M
Age(yr)	8	2.5	5.5	5	19	9
Age at onset	10 days	At birth	1 mon	9 mon	since birth	4 mon
Age at diagnosis	4 mon	3 mon	4 mon	1 year	3 mon	5 mon
Initial presentations	-Diarrhea -Lethargy -Hypoglycemia -Hepatomegaly -Failure to thrive -Poor feeding -Metabolic acidosis	-Respiratory distress -Vomiting -Hypoglycemia -Metabolic acidosis -Poor feeding	-Aphthous stomatitis -Pneumonia -Diarrhea -Cellulite around the breast -Hepatomegaly	-Hypoglycemia -Hepatomegaly	-Hepatomegaly -Nausea -Acidosis -Poor feeding -Elevated triglyceride and cholesterol	-Metabolic acidosis -Lethargy -Tachypnea -Vomiting -Poor feeding -Seizure at birth -Hepatomegaly
Liver biopsy	-Liver steatosis	-Compatible with GSD	-Liver steatosis	-Liver steatosis	-Liver steatosis	-Liver steatosis
Infections	-Otitis -Respiratory tract infection -Gastroenteritis -Sever aphthous stomatitis	-Aphthous stomatitis -Pneumonia	-Recurrent pneumonia -Oral candidiasis -Aphthous stomatitis -Nipple abscess	-Otitis media -Gingivitis	-Otitis -Pharyngitis -Periodic aphthous stomatitis	-Aphthous stomatitis
Neutropenia^a^						
WBC (*10^3^/μl)	7.3	6.99	6.0	7.08	1.5	2.5
ANC /μL	400	489	300	977	267	500
Hepatomegaly	+	+	+	+	+	+
Hypoglycemia ( <60mg/dl)	+	+	+	+	+	+
Chubby face	+	+	+	+	-	-
Increased AST/ ALT (Up to 37 U/L)/ (Up to 41 U/L)	+	+	+	+	-	-
Hyperlipidemia (>160 mg/dl)	+	+	+	+	-	-
Hypercholesterolemia (>200 mg/dl)	-	-	+	-	+	-
Hyperlactatemia (>2.5 mg/dl)	+	+	+	+	NA	NA
Hyperuricemia (>5 mg/dl)	+	+	-	+	-	-
Other features	-Nephromegaly -IBD-Like disorder - Anemia -Hospitalization (12 times)	-Hospitalization (4 times) -Increased ESR -Anemia -Seizure (one time) -Presence atypical cells in peripheral blood smear (PBS)^b^	-Hepatosplenomegaly -Increased ESR -Epistaxis -Seizure (4 times) -Severe hearing loss -Otitis -Autism-like behaviors - Vision weakness - Strabismus -Developmental delay -Gingivitis - Anemia	-Hypothyroidism	-Nephromegaly -Increased ESR -Anemia -Hepatosplenomegaly	-Increased ESR -Anemia -Severeosteopenia
Prescribed Drugs	-Corn starch -Allopurinol -Bicarbonate -G-CSF -Intravenous injection of Mg, P and Na solutions -Phenobarbital -Mupirocin -Fluconazol	-Corn starch -Galactomin -G-CSF -Allopurinol -Bicarbonate	-Corn starch -G-CSF -Anti-seizure drugs	-Corn starch -Bicarbonate	-Corn starch -G-CSF	-Corn starch -G-CSF

P4 showed hypoglycemia and hepatomegaly in the first year of life. Laboratory examinations showed elevated triglyceride (TG) concentration and uric acid but liver transaminases were normal to slightly increased. Development, growth and facial appearance were normal. Liver biopsy showed severe fatty changes (microvesicular and macrovesicular) and steatosis. The liver was enlarged with normal echogenicity and both kidneys are mildly enlarged. Furthermore, she presented clinically with otitis media, gingivitis, neutropenia (WBC = 7.08*10^3^/μl, Neutrophil count =966) without leukopenia. Her twin sister had almost similar clinical presentations such as hypoglycemia and hepatomegaly and presented steatosis in liver biopsy. However, she suffered from congenital cataract at the first month of life, growth retardation, epistaxis and elevated creatine phosphokinase (CPK) (data not available) and finally she died due to hypoglycemic coma after 1 year without a definite diagnosis.

Patient P5–1, a 19-year-old girl had suffered from severe neutropenia with leukopenia (WBC = 1.7*10^3^/μl, Neutrophil count = 289/μl) that in the first days of life referred to our pediatric clinic with nausea, acidosis, poor feeding, elevated TG (218 mg/dl), hypoglycemia and hepatomegaly. At sampling date, laboratory examination revealed normal fasting blood glucose, uric acid, TG, and liver transaminases. She had recurrent infectious with otitis, pharyngitis, periodic aphthous stomatitis. Other clinical observations were mild hepatosplenomegaly, enlarged bilateral kidneys, anemia, and increased Erythrocyte Sedimentation Rate (ESR). Chest X-Ray showed bilateral reticular infiltration and decreased bone density. The result of liver biopsy revealed ballooning changes with feathery degeneration and mild steatosis. She had a brother, patient P5–2, a 9-year old boy with similar clinical and laboratory manifestations. He was brought to the pediatric clinic due to seizure at birth, an elevated level of TG concentration (464 mg/dl) and moreover, at 4 months, he suffered from severe metabolic acidosis, lethargy, tachypnea, fever, vomiting, poor feeding, and hepatomegaly. Also, he experienced recurrent aphthous stomatitis, neutropenia with leukopenia (WBC = 2.5*10^3^/μl, Neutrophil count = 570/μl), increased ESR, anemia and severe osteopenia. He was the second child of the consanguineous family with no family history of GSD.

### Molecular analysis of the identified variants

From the twenty studied patients, six showed autozygous haplotypes for the STR markers flanking the *SLC37A4* gene and any patients did not show any autozygosity for the markers flanking the *G6PC* gene. Sequence analysis of the entire and intron/exon boundaries of the *SLC37A4* gene revealed three different mutations in patients with a homozygous haplotype. Haplotype map was shown for families with novel mutations in Fig. 1. Three patients showed two novel mutations (c.365G > A (p.G122E) and whole *SLC37A4* gene deletion) and three patients presented a recurrent mutation (c.1042_1043del (p.Leu348Valfs*53)). The recognized mutations in patients were homozygous and were not present in their healthy members of the family. Segregation analysis showed that their parents were heterozygous for the recognized mutations.

#### The first novel mutation, c.365G > A (p.G122E) in the 4th exon

**Fig. 2:**
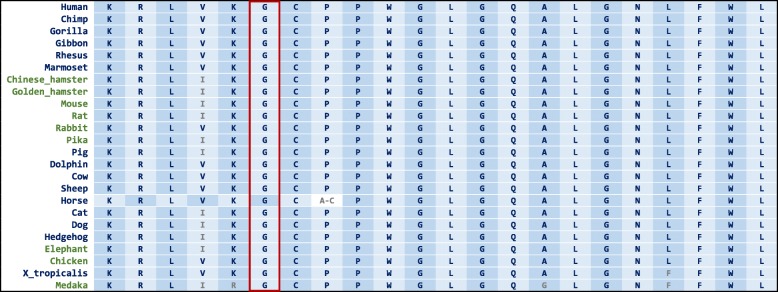
Multispecies alignment for the identified variant in P4 patient: c.365G > A, p.G122E. The panel from the UCSC genome browser (https://genome.ucsc.edu/cgi-bin/)

One of the two novel variants, c.365G > A (p.G122E) in 4th exon, was identified in the P4 patient that was homozygous for this variant. The twin sister died and her DNA was not available. According to our survey, the variant was not found in any of the population or disease databases as mentioned above. In silico predictive tools showed the deleterious effect on the variant on gene product (Table 3). The evolutionary tools showed glycine residue at the position 122 of *SLC37A4* protein is highly conserved (PhyloP, PhastCons, and GERP; 4.3 and 1.0 and 5.1, respectively) and multispecies alignment is shown for this variant in UCSC genome browser (Fig. 2). As predicted by the HOPE project, the mutant residue is bigger and less hydrophobic than the wild-type residue and is negatively charged. According to Varsome tool, this variant is predicted to be variant of uncertain significance (VUS) based on these evidences: (1) The absence of detected variant in any of the population databases (PM2). (2) Missense variant in the *SLC37A4* gene that has a low rate of benign missense variation and in which missense variants are a common mechanism of GSD1b disease (PP2) and multiple bioinformatics evidence support a deleterious effect on the gene or the protein (PP3).

**Table 3:** Table caption

	Patients	P1, P2 and P3	P4	P5-1 & P5-2
Variant Definition	*Varian name* *Protein Change*	*c.1042_1043delCT* p.Leu348Valfs*53	*c.365G>A* *G122E*	*g.118895235_118901946del6712*
	*Chromosome Position (GRCh37)* *Zygosity*	*Chr11 :118895981_118895982* *Homozygote*	*Chr11: 118898920* *Homozygote*	*Chr11: 118895235 to chr11: 118901946* *Homozygote*
*In-silico* Predictive Tools	*CADD (Phred Score)* *DANN* *Polyphen-2* *PROVEAN*	*35(Deleterious)* *-* *-* *-*	*27.4 (Deleterious)* *0.82 (Deleterious)* *0.958 (PROBABLYDAMAGING)* *2.66 (Deleterious)*	*-* *-* *-* *-*
Population/Disease Databases	*1000 GP* *ExAC* *GenomAD* *ESP* *HGMD* *Clinvar*	*-* *0.00023* *0.00018* *0.0003* *CD982664* *VCV000006926*	*-* *-* *-* *-* *-* *-*	*-* *-* *-* *-* *-* *-*

#### The second novel mutation, SLC37A4 gene deletion

**Fig. 3:**
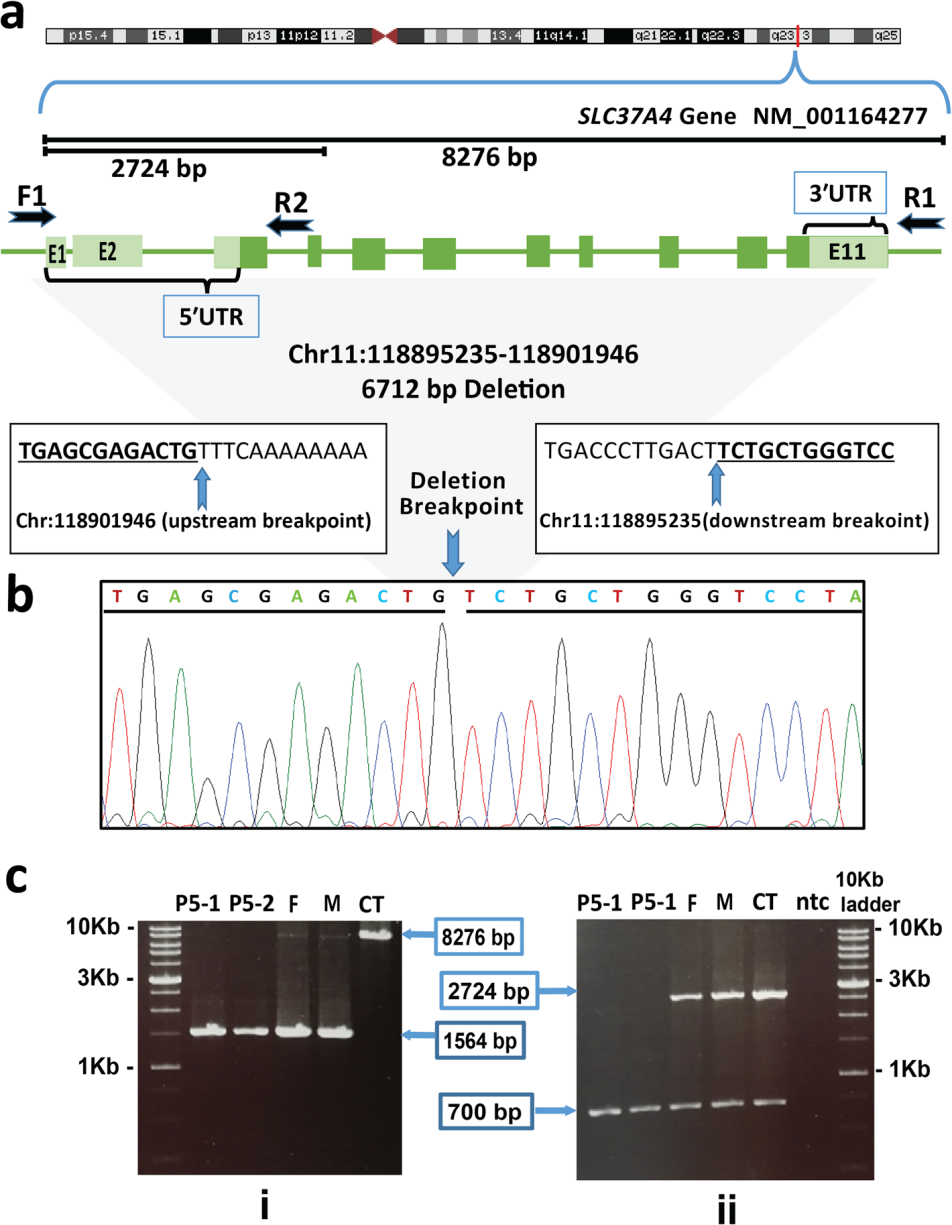
Long-range PCR and sequencing showed the full-gene deletion of *SLC37A4* in the siblings (P5–1 and P5–2) with GSD1b. **a** the gene transcript image (was taken from Genome Data Viewer in NCBI) and the orientation of the designed primers across upstream and downstream breakpoint. The black arrows indicate the position of primers used in Long-range PCR. The length of the genomic segment for each set of primers (F1&R1 and F1&R2) was shown. **b** Sanger sequencing result of the breakpoint site and flanking region. Two boxes above the sequencing result indicate the sequences across upstream and downstream breakpoint. In Sanger sequencing result, the blue arrow shows the breakpoint and a 6712 bp sequence deletion on chr11 of the human reference genome (GRCh37). **c** Gel electrophoresis of the PCR product. i) Results of the first long-range PCR (PCR with F1 and R1 primers) are presented in the left which shows this segment in the siblings, parents and control samples. Lane 1 contains a 10 kb ladder, lane 2 and 3 contains the products of deleted allele, lane 3 and 4 contains the products of both the deleted allele and the wide type. Lane 5 contains the wide-type allele. ii) Results of the second long-range PCR (PCR with F1 and R2) are presented in the right which shows this segment in two siblings, parents and control samples. Lane 1 contains a 10 kb ladder, lane 2 contains NTC, Lane 3, 4 and 5 contains a 2724 bp fragment without deletion and lane 6 and 7 contains no amplification. All lanes (except lane 2) include a ~ 700 bp internal control (Exon 5 of *G6PC* gene). The products of deleted allele, (1564 bp); the products of wide-type allele, (8276 bp); M, mother; F, father; CT, control sample

In the sibling (P5–1 and P5–2), a systematic failure to amplify genomic DNA covering all exons of the *SLC37A4* gene directed the possible presence of whole gene deletion (g.118895235_118901946del6712 (GRCh37)). To confirm the presence of full-gene deletion and finding the exact breakpoint site, primer walking with the help of three primers (F1, R1, and R2) was done (Table 1). For the first Long-range PCR assay, F1 and R1 primers were designed flanking regions of the suspected deletion (876 bp upstream and 844 bp downstream of the *SLC37A4* gene). Sanger sequencing was performed to identify the exact location of the breakpoint site. The results of sequencing established our prediction and identified the location of breakpoint precisely at Chr11:118895235–118,901,946 (Fig. 3). It permitted us to determine the exact size of the deleted region (6712 bp). Gel electrophoresis of this PCR product revealed homozygote deletion in the sibling and heterozygote deletion in parents. The size of DNA fragment in siblings was 1564 bp, while the size of the expected genomic segments without deletion was 8276 bp in the control sample, and both segments exist in parents as expected. To further confirm the presence of deletion, the second Long-range PCR assay with another set of primers (F1 and R2) was used to discriminate between cases with and without deletion. R2 primer was designed around the 4th exon as an internal control. To visually confirm the mutant and wide type alleles, PCR products were run on gel electrophoresis and generated a 2724 bp fragment in parents. There was no amplification in patients bearing the deletion.

## Discussion

GSD1 is the most prevalent hepatic type of Glycogen storage diseases (GSDs) which comprises a group of autosomal recessive disorders characterized by the deficiency of the enzymes that regulate the synthesis or degradation of glycogen. GSD1 categorized into two overlapping forms, 1a and 1b which caused by the deficiency of *G6PC* and *SLC37A4* genes, respectively [1]. The high rate of consanguineous marriage in Iran proposes a high incidence of autosomal recessive disorders. Since GSD1 is a rather rare disease in Iran and other populations, only a few studies have been published regarding the GSD1 mutational spectrum in Iran and elsewhere [21], so we decided to conduct autozygosity mapping to quick and indirectly find the mutated gene in nineteen families suspected to GSD. Haplotype analysis of the studied families showed six patients with autozygous haplotype block for the *SLC37A4* gene and no family was autozygous for the markers flanking the *G6PC* gene. Subsequently, the sequencing of the *SLC37A4* gene was revealed two novel and one recurrent mutation in six patients. This is the first study to summarize the clinical and molecular characteristics of Iranian patients with GSD Ib.

*SLC37A4* deficiencies affect metabolic and myeloid phenotypes. In metabolic phenotype, in gluconeogenic organs of liver, kidney, and intestine, *SLC37A4* and *G6PC* together are required to maintain interprandial blood glucose homeostasis. In myeloid phenotype, *SLC37A4* and *G6PC3* together are required to maintain neutrophil homeostasis and their deficiency leads to immune deficiency, characterized by neutropenia and neutrophil dysfunction [3]. Previous Studies have shown that GSD-Ib patients exhibit an increased risk of developing autoimmune disorders, including IBD, thyroid autoimmunity, and myasthenia gravis. Melis and colleagues exhibited that GSD-Ib patients indicated lymphopenia and T cells exhibit altered glycolysis and impaired peripheral regulatory T cell function [22]. In previous studies, the most common mutation was c.1042_1043del (p.Leu348Valfs*53) that has been recurrently reported in German (32%) populations and mixed Caucasian (27–31%) [23]. The mentioned deletion in the 8th exon leads to the deficiency of enzyme activity which accompanied by severe neutropenia in three patients in our study (P1, P2, and P3). These three patients have several hospitalizations due to hypoglycemia and seizure attacks. In addition to GSD1b common symptoms (OMIM:232220), P3 suffered from secondary symptoms such as vision weakness, severe hearing loss, strabismus, developmental delay and autism-like behaviors that had not been reported with this disease in previous publications. These signs may be due to recurrent severe seizures.

Regarding the novel c.365G > A (p.G122E) mutation in P4 patient, glycine at the transmembrane domain of the glucose 6-phosphate translocase enzyme is more hydrophobic than the mutant residue. This difference in hydrophobicity can affect the hydrophobic interactions with the membrane lipids. Furthermore, glycine is the most flexible of all residues. This flexibility might be necessary for the protein’s function [14]. Mutation of this glycine can abolish this function and coincidently, the torsion angles of this residue are incorrect. In silico analysis tools were consistent in predicting that this variant may impair protein function and this substitution can change the enzyme conformation. The hematological findings in this patient showed moderate neutropenia in contrast to P1, P2, and P3 patients (frameshift variant) with severe neutropenia. The CADD score, which has high sensitivity to predict molecular pathogenicity of variants, was 27 and 35 in missense variant (p.G122E) and frameshift variant (p.Leu348Valfs*53), respectively. Since the higher values of CADD score predict more severe effect, it may explain the pronounced hematological finding in P1, P2, and P3 patients in comparison to P4.

The second novel mutation, whole *SLC37A4* gene deletion, was identified in a sibling (P5–1 and P5–2). The homozygote 6.7 kb deletion which spans near the entire *SLC37A4* gene can lead to the complete loss of function of both alleles. Therefore, G6PT protein will not be produced. To date, 116 mutations were identified for *SLC37A4* gene (http://www.hgmd.org) including 86 substitutions and 30 small/gross deletion and insertions. To the best of our knowledge, this is the first report of whole *SLC37A4* gene deletion. Here, the siblings (P5–1 and P5–2) had anemia, leucopenia, and severe neutropenia with increased lymphocyte counts. These hematological findings suggest a correlation between entire *SLC37A4* gene deletion and severe myeloid problems in GSD-Ib.

However, in previous studies, no correlation was reported between individual mutations and present/absence of neutropenia, bacterial infections, and other complications [24–26]. Recently, one study by Sarajlija A et al., revealed some specific mutations in *SLC37A4* have an impact on the severity of neutropenia and the capacity of increasing neutrophil count in serious bacterial infections (SBI) [27]. It is worth to mention that all GSD1b patients did not have neutropenia and some of them may suffer from cyclic neutropenia [5]. This phenotypic variability of GSD 1b might suggest contribution of one or several other factors (ie., as modifier genes) in phenotype disease, which could change “simple” Mendelian disorders into complex traits [28].

## Conclusions

Clinical and biochemical parameters were different in the GSD1b patients. Therefore, the strict genotype-phenotype correlation couldn’t be demonstrated based on this data. However, hematological findings revealed a correlation between causative mutations and myeloid phenotypes. P1, P2, and P3 with frameshift variant (p.Leu348Valfs*53) had severe neutropenia. P4 with a missense mutation (p.G122E) showed moderate neutropenia. The sibling (P5–1 and P5–2) with whole *SLC37A4* deletion represented both severe neutropenia and leukopenia. This large deletion was accompanied by severe impairment of myeloid cells. But establishing a definitive genotype-phenotype correlation would needs more studies with larger sample sizes. Since GSD is a group of clinically and genetically heterogeneous disorders, it is recommended to use whole-exome sequencing (WES) for the detection of causative mutations in families with no autozygous haplotype block for the markers flanking the *G6PC* gene.

## Data Availability

The datasets are available from the corresponding author on reasonable request.

## Abbreviations

1000 GP: 1000 genome project
ALT: Alanine transaminase
AST: Aspartate aminotransferase
CADD: Combined Annotation Dependent Depletion
CPK: Creatine phosphokinase
ESP: Exome Sequencing Project
ESR: Erythrocyte Sedimentation Rate
ExAC: Exome Aggregation Consortium
G6Pase-α: Glucose-6-phosphatase-α
G6PT: Glucose-6-phosphate transporter
GSD: Glycogen storage disease
HGVS: Human Genome Variation Society
IBD: Inflammatory bowel disease
SBI: Serious bacterial infections
SERV: Sequence-based Estimation of Repeat Variability
STR: Short tandem repeat
TG: Triglyceride
TRF: Tandem Repeat Finder
VUS: Variant of uncertain significance
WBC: White blood cell
WES: Whole-exome sequencing
